# Self-rated work ability predicts return to work after occupational rehabilitation

**DOI:** 10.3389/fresc.2026.1731625

**Published:** 2026-02-26

**Authors:** Chris Jensen, Tarja Rajalahti Kvalheim, Svein Ove Tjøsvoll, Anita Dyb Linge, Thomas Johansen

**Affiliations:** 1 Rehabiliteringssenteret AiR, Rauland, Norway; 2 Red Cross Haugland Rehabilitation Centre, Flekke, Norway; 3 Betania Malvik Avdeling Helse og Arbeidsliv, Malvik, Norway; 4 Muritunet Rehabilitation Centre, Valldal, Norway; 5 Department of Social Science and History, Volda University College, Volda, Norway; 6 Hernes Occupational Rehabilitation Centre, Hernes, Norway

**Keywords:** occupational rehabilitation, physical medicine and rehabilitation, return to work, work ability, work participation

## Abstract

**Introduction:**

Work ability assessments are important for planning, targeting, and executing occupational rehabilitation programs. The aim of the study was to examine whether a one-item self-rated Work Ability Score (WAS), assessed before and after rehabilitation, predicts return to work 12 months after occupational rehabilitation.

**Methods:**

Data from four institutions in the *Norwegian registry for occupational rehabilitation* were used. This cohort study included participants with a history of sick leave and complex musculoskeletal and mental health-related challenges who had participated in an occupational rehabilitation program, with a 12-month follow-up period. WAS was self-reported on a scale from 0 to 10 at the start and end of rehabilitation and linked to registry data on health-related social income benefits, as well as self-reported sociodemographic variables. Logistic regression analyses were conducted with “full return to work” (full RTW), defined as receiving no health-related social security benefits for 1 month) at 12 months follow-up as an outcome.

**Results:**

Higher WAS values were positively associated with full RTW. The odds ratio for a one-point increase in WAS at the start of rehabilitation was 1.57 [95% confidence interval (CI): 1.41–1.75], and it was 1.47 (95% CI: 1.33–1.63) for a one-point increase in improvement of WAS from start to end of rehabilitation when controlling for age, sex, education, occupational status and benefits received before rehabilitation.

**Conclusion:**

Even a one-point increase in WAS significantly improved the odds of future work participation. Designing rehabilitation programs to target the workability of participants may initiate a process that leads to work participation.

## Introduction

Norway has the highest level of sick leave compared with other Nordic and European countries (1). Participation in work provides a gateway to community involvement, enabling individuals to contribute to society. It also offers social interaction, opportunities for personal growth, and confers identity, status, meaning, and purpose in life. Therefore, there are strong individual, economic, social, and moral reasons to reduce long-term sick leave and facilitate workplace inclusion.

Norway has a universal health coverage system funded through taxation. All employees are also covered by a mandatory sickness insurance scheme that provides full income compensation for up to 52 weeks in the event of illness or injury. The first 16 days are paid by the employer, whereas income during the remaining period is reimbursed by the state. After 52 weeks, individuals with reduced work ability may apply for a work assessment allowance, which compensates approximately two-thirds of their previous income and is also state-funded. While receiving benefits, individuals are required to participate actively in activities aimed at clarifying and improving their work capacity. This involves collaboration with case workers within the social security system to develop and follow plans for return to work (RTW). Work assessment allowance can be granted for a maximum duration of 3 years, with the possibility of extension for up to 2 additional years.

Nationwide, public, non-profit, and private actors run rehabilitation centers within the specialist healthcare service in agreement with the four regional health authorities. To address health and functional capacity issues related to returning to work, individuals in Norway who struggle to remain employed can receive several interventions, including occupational rehabilitation. Occupational rehabilitation aims to encourage and facilitate work participation despite health complaints and may improve work ability and facilitate earlier RTW after sick leave (2–6).

Participation in an occupational rehabilitation program in the specialist healthcare service requires referral from a medical doctor. Occupational rehabilitation is a multidisciplinary intervention intended to assist sick-listed employees in returning to and maintaining employment. It can be delivered either as an intensive day program or as an inpatient program lasting several weeks. Such programs are part of a collaborative chain in the follow-up of sick-listed employees, in which the workplace and occupational health service, the general practitioner/treating physician, and the case worker in the social security system may be involved before, during, and after the program. Occupational rehabilitation within the specialist healthcare setting is typically reserved for a limited subset of employees receiving benefits, namely, those with complex work and health challenges that impede work participation. When referred, the participants must have tried other simpler interventions, their health problems must have been sufficiently treated to allow participation in rehabilitation programs, and that participants must agree on work participation as the main goal of rehabilitation. Occupational rehabilitation programs employ multidisciplinary approaches to address the complex interplay of factors hindering work participation. Rehabilitation teams usually consist of a physician, a psychologist, a physiotherapist, a sports pedagog, and a work consultant. External stakeholders, such as the general practitioners, employers, or case workers, are involved as needed.

Work participation necessitates adequate work ability, a key concept in occupational rehabilitation (7, 8). Within the framework of the International Classification of Functioning, Disability and Health (ICF), work ability refers to the functional capacity to perform work and arises from the interaction among individual factors, such as physical, mental, and social factors, and contextual and environmental factors. Thus, assessing work ability should acknowledge its multidimensional nature (9, 10). Work ability balances a worker's resources, demands, and opportunities, all of which are influenced by the external environment (9, 11). Tengland (12) defined *specific work ability* as the manual, intellectual, and social competences, together with the physical, mental, and social health, required to perform a specific work. Furthermore, *General work ability* may be defined similarly with respect to work in general. Previous studies have recognized that work ability is a complex concept and that its exact definition remains somewhat unclear. In addition, there is ambiguity regarding how the assessment of work ability predicts an individual's future capabilities and the expected duration of sick leave (13, 14).

The current study will advance the understanding of the association between self-rated work ability and the use of health-related social security benefits among sick-listed workers participating in occupational rehabilitation. The growth of work-oriented programs in Norway requires the development of evidence-based standards and guidelines on the use of work ability measures, both in the initial stage to assess and monitor progress in work ability and in the final stage to monitor goal achievement. Moreover, identifying factors beyond sufficient levels of work ability that contribute to RTW is highly relevant for clinicians to better determine the prognosis of sick-listed workers and to design individual rehabilitation plans.

Education level, socioeconomic status, and employment status are among the other factors that may influence the chance of RTW of participants in rehabilitation programs (15, 16).

A frequently used instrument for assessing work ability is the Work Ability Index (WAI), developed by Ilmarinen (17) at the Finnish Institute of Occupational Health. In epidemiological studies, a single-item question, the Work Ability Score (WAS), from WAI has been shown to predict sickness absence (11, 18, 19). Also, in clinical work, a single-item score is preferable for assessing work ability.

Ståhl et al. (13) reported that WAS was the only predictor of future sick leave in a study of more elaborate work ability assessments of sick-listed individuals within the Swedish sickness insurance scheme. They combined both objective and self-reported assessments of general work ability to evaluate their predictive value. The main aim of these assessments was to determine eligibility for social security benefits. In a large register-based Finnish study, WAS was a strong predictor of long-term sick leave and disability pension (20). Thus, it seemed promising to use WAS for assessing work ability in occupational rehabilitation, but this context differs from those in previous studies, which may affect its predictive value. Self-reports of work ability may vary depending on the aim of the assessment, i.e., whether the aim is to ensure eligibility for benefits or to assess rehabilitation needs. Furthermore, compared with participants in large population studies, individuals undergoing rehabilitation are characterized by poor work ability, and it therefore remains unclear whether small differences in WAS have significant predictive value for future work participation.

Hence, the main aim of the present study was to examine the one-item self-rated WAS before and after rehabilitation as a predictor for RTW 12 months after occupational rehabilitation.

## Methods

### Study design

This is a multicenter registry-based retrospective cohort study with a 12-month follow-up.

Data from the *Norwegian registry for occupational rehabilitation* were used. The registry collects data and documents changes in work participation and self-assessed work ability from participants receiving occupational rehabilitation in Norway. The participants provided written informed consent and completed a 15-item questionnaire, including the WAS, before rehabilitation. Immediately after rehabilitation, work ability was reassessed.

All institutions that offer occupational rehabilitation in Norway can participate in the registry. Invitations to participate and data collection through questionnaires were conducted at each participating institution. Questionnaires responses were linked to data on payments of health-related social security benefits for each participant during the 12 months following occupational rehabilitation.

### Study sample

The participants in the present study received occupational rehabilitation at four institutions in 2022, located in two health regions. The services were mainly provided through inpatient rehabilitation programs lasting ≥4 weeks, but at one institution, an intensive outpatient program was delivered. All programs were offered by interdisciplinary teams and contained both health- and work-oriented interventions. Participants were referred to rehabilitation from general practitioners due to a history of sick leave and complex challenges related to health issues and work participation. The occupational rehabilitation programs were transdiagnostic, meaning they were not designed for specific diagnoses. However, the majority of long-term absentees were on sick leave due to musculoskeletal conditions, common mental disorders (primarily anxiety and depressive disorders), fatigue, stress-related symptoms, and other mental and physical challenges that negatively affected their work ability.

### Variables

#### Sociodemographics

Self-reported individual characteristics and assessed at the beginning of rehabilitation in the present study were age, sex, level of education, occupational status, and job type.

Education was categorized based on type of education and number of years completed: (1) elementary school <10 years, (2) vocational education, (3) high school, (4) supplementary education (vocational + 2 years of supplementary education), (5) bachelor's degree, and (6) master's degree.

#### Work Ability Score

WAS was assessed as the current work ability relative to the lifetime best, on a scale from 0 (lowest possible) to 10 (lifetime best) points. It is widely used at the beginning and end of occupational rehabilitation programs in Norway, and it has also been used in other rehabilitation programs (21). Here, WAS was measured at the start and end of rehabilitation, and the change from start to end of rehabilitation was calculated.

#### Work participation

From the Norwegian National Social Security System Registry, payment of health-related social security benefits was obtained from the start of rehabilitation and for the 12 subsequent months, with data collected every month. In the present study, benefits were observed for 1 full month during rehabilitation and for an entire month 1 year after rehabilitation. The outcome “full return to work” (full RTW) was defined as not receiving any health-related social security benefits for an entire month 1 year after rehabilitation.

### Statistical analysis

Descriptive statistics were presented as frequencies for categorical variables or mean values and standard deviations for continuous variables. Differences between the groups were examined using the *χ*^2^ test or Fisher's exact test for categorical variables and using Student's *t*-test or one-way ANOVA for continuous variables.

The binary outcome “full RTW” was used in multivariate logistic regression analyses, with results presented as odds ratios (ORs) and corresponding 95% confidence intervals (95% CIs). The analyses estimated odds ratios for education, employment status, receipt of benefits during rehabilitation, and self-assessed work ability measured before and after rehabilitation. Regression analyses were adjusted for age, sex, and the other variables mentioned above.

Analyses were carried out using StataNow 18.5, Standard Edition (StataCorp LLC, Texas, USA).

### Ethical considerations

All participants received oral and written information about the Register and signed an informed consent form in accordance with the Declaration of Helsinki.

Establishment of the Register was approved by the Norwegian Data Protection Authority (16/01688-2/RHY).

## Results

The Norwegian register on occupational rehabilitation contained information from 907 individuals in 2022. Only four individuals did not respond to any items in the questionnaire. Demographic characteristics were unevenly distributed between women (*n* = 642) and men (*n* = 261, Table 1). Men were relatively more represented in both the youngest and oldest age groups and had shorter educational attainment than women. Mean age was similar between sexes: 46.0 (SD = 10.1) for women and 45.3 (SD = 12.1) for men. A higher proportion of men were unemployed and received work assessment allowance compared with women. The mean WAS for all respondents increased from 3.3 at the start of rehabilitation to 4.0 at the end, with no significantly differences in scores between women and men (Table 2). However, the mean difference in WAS from the start to the end of rehabilitation was significantly higher for men (mean difference = 1.0) than for women (mean difference = 0.6).

**Table 1:** Sociodemographics.

Variable	Women	Men	*p*-Value^a^
	*N* = 642	*N* = 261	
	*n* (%)	*n* (%)	
Age (years)			**0** **.** **002**
18–29	52 (8.1)	41 (15.7)	
30–39	119 (18.5)	38 (14.6)	
40–49	193 (30.0)	60 (23.0)	
50–59	232 (36.1)	96 (36.8)	
60–69	46 (7.2)	26 (10.0)	
Missing	0 (0)	0 (0)	
Education			**<0**.**001**
10 years or less	50 (7.8)	52 (19.9)	
Vocational education	158 (24.6)	98 (37.5)	
High school	69 (10.7)	31 (11.9)	
Supplementary education	29 (4.5)	18 (6.9)	
Bachelor	249 (38.8)	45 (17.2)	
Master	73 (11.4)	8 (3.1)	
Missing	14 (2.2)	9 (3.4)	
Most recent job			**<0**.**001**
Unskilled work or training	36 (5.6)	26 (10.0)	
Skilled, manual work	57 (8.9)	87 (33.3)	
Office or service work	177 (27.6)	29 (11.1)	
Health or school work	188 (29.3)	28 (10.7)	
Academics	48 (7.5)	9 (3.4)	
Managers	47 (7.3)	26 (10.0)	
Other	74 (11.5)	47 (18.0)	
Missing	15 (2.3)	9 (3.4)	
Occupational status			**0**.**001**
Employed	533 (83.0)	190 (72.8)	
Unemployed	94 (14.7)	62 (23.8)	
Missing	15 (2.3)	9 (3.4)	
Benefits during rehabilitation			**0**.**017**
Partial sick leave benefits	215 (33.5)	65 (24.9)	
Full sick leave benefits	181 (28.2)	77 (29.5)	
Work assessment allowance	180 (28.0)	97 (37.2)	
Other	66 (10.3)	22 (8.4)	
Missing	0 (0)	0 (0)	

Statistically significant values are bold.

a *χ*^2^ test or Fisher's exact test for assessing differences between women and men.

**Table 2:** Work ability and gender.

WAS	Total	Women	Men	*p*-Value^a^
	Mean (SD)	Mean (SD)	Mean (SD)	
Before rehabilitation	3.3 (2.3)	3.3 (2.3)	3.1 (2.1)	0.30
After rehabilitation	4.0 (2.6)	4.0 (2.7)	4.1 (2.7)	0.50
Difference	0.7 (2.1)	0.6 (2.1)	1.0 (2.1)	**0** **.** **01**

Statistically significant values are bold.

a *t*-Test for assessing differencing between women and men.

Full RTW 12 months after rehabilitation was more often achieved by participants with vocational education or a higher education, those who were employed, and those on partial sick leave (Table 3). Mean WAS values at the start and end of rehabilitation were significantly higher among participants who achieved full RTW compared with those who continued to receive social security benefits 12 months after rehabilitation (Table 4). The mean difference in WAS between the start and end of rehabilitation was 1.4 among those who achieved Full RTW, and the mean WAS at the end of rehabilitation was 5.8. A relatively large group of respondents reported lower WAS at the end of rehabilitation compared to the start. Among those who did not return to work, 23% reported a decrease in WAS, 32% reported the same score, and 45% reported an increase in WAS at the end of rehabilitation. Among those who returned to work 14% reported a decrease in WAS, 19% reported the same score, and 67% reported an increase in WAS at the end of rehabilitation.

**Table 3:** Demographics and full RTW (receiving no benefits 12 months after rehabilitation).

Variable	Full RTW		*p*-Value^a^
	Yes	No	
	*n* (%)	*n* (%)	
Age (years)			0.99
18–29	29 (31.2)	64 (68.8)	
30–39	46 (29.1)	112 (70.9)	
40–49	78 (30.6)	177 (69.4)	
50–59	97 (29.5)	232 (70.5)	
60–69	22 (30.6)	50 (69.4)	
Total	272 (30.0)	635 (70.0)	
Sex			0.59
Female	190 (29.6)	452 (70.4)	
Male	82 (31.4)	179 (68.6)	
Total	272 (30.1)	631 (69.9)	
Education			**<0** **.** **001**
10 years or less	14 (13.7)	88 (86.3)	
Vocational education	95 (37.1)	161 (62.9)	
High school	21 (21.0)	79 (79.0)	
Supplementary education	14 (29.8)	33 (70.2)	
Bachelor	96 (32.7)	198 (67.4)	
Master	26 (32.1)	55 (67.9)	
Total	266 (30.2)	614 (69.8)	
Most recent job			0.32
Unskilled work or training	13 (21.0)	49 (79.0)	
Skilled, manual work	46 (31.9)	98 (68.1)	
Office or service work	63 (30.6)	143 (69.4)	
Nurses, teachers, etc.	66 (30.6)	150 (69.4)	
Academics	17 (29.8)	40 (70.2)	
Managers	30 (41.1)	43 (58.9)	
Other	31 (25.6)	90 (74.4)	
Total	266 (30.3)	613 (69.7)	
Occupational status			**<0** **.** **001**
Employed	241 (33.3)	482 (66.7)	
Unemployed	24 (15.4)	132 (84.6)	
Total	265 (30.2)	614 (69.8)	
Benefits during rehabilitation			**<0** **.** **001**
Partial sick leave	147 (52.5)	133 (47.5)	
Full sick leave	91 (35.0)	169 (65.0)	
Work assessment allowance	15 (5.4)	264 (94.6)	
Other	19 (21.6)	69 (78.4)	
Total	272 (30.0)	635 (70.0)	

Statistically significant values are bold.

a *χ*^2^ test or Fisher's exact test.

**Table 4:** Work ability and full RTW (receiving no benefits 12 months after rehabilitation).

WAS	Full RTW	No full RTW	*p*-Value^a^
	Mean (SD)	Mean (SD)	
Before rehabilitation	4.5 (2.1)	2.8 (2.1)	**<0** **.** **001**
After rehabilitation	5.8 (2.3)	3.2 (2.3)	**<0**.**001**
Difference	1.4 (1.9)	0.5 (2.1)	**<0**.**001**

Statistically significant values are bold.

a *t*-Test.

WAS also differed between participants who received different social security benefits during rehabilitation (Table 5). The lowest score was consistently reported by those who received work assessment allowance, and they also reported a lower increase in WAS from the start to the end of rehabilitation than those receiving partial or full sick leave benefits during rehabilitation.

**Table 5:** Work ability and health-related social security benefits during rehabilitation.

WAS	Sick leave benefits				*p*-Value^b^
	Partial	Full	WAA^a^	Other	
	Mean (SD)	Mean (SD)	Mean (SD)	Mean (SD)	
Before rehabilitation	4.7 (1.9)	2.6 (2.1)	2.6 (2.1)	3.1 (2.2)	**<0** **.** **001**
After rehabilitation	5.4 (2.4)	3.7 (2.5)	2.9 (2.2)	3.8 (2.6)	**<0**.**001**
Difference	0.8 (2.0)	1.1 (2.3)	0.3 (1.9)	0.7 (2.1)	**<0**.**001**

Statistically significant values are bold.

a Work assessment allowance.

b One-way ANOVA.

In multivariable regression analyses, full RTW was predicted by education level, benefits received during rehabilitation, and WAS (Table 6). Both vocational education and higher education levels strongly predicted full RTW, even after adjustment for age, sex, and other variables in the models shown in Table 6. Benefits received during rehabilitation were consistently the strongest predictor of Full RTW, as sick leave beneficiaries had markedly higher chances of full RTW than those who received work assessment allowance. Occupational status was included in the model but was not a significant predictor of full RTW due to collinearity with benefits received during rehabilitation. Different models including the WAS were calculated. In model 4, both WAS at the start of rehabilitation (OR = 1.57) and the difference in WAS from the start to the end of rehabilitation (OR = 1.47) were included.

**Table 6:** Work ability score (WAS) as a predictor of full RTW (receiving no benefits at 12 months follow-up).

Variables	Model 1	Model 2	Model 3	Model 4
	OR (95% CI)	OR (95% CI)	OR (95% CI)	OR (95% CI)
Education				
<10 years	1		1	
Vocational education	**3.64** **(1.86–7.13)**	**3.69** (**1.78–7.61)**	**4.22** (**1.92–9.26)**	**4.16** (**1.89–9.16)**
High school	1.52 (0.68–3.39)	1.73 (0.74–4.05)	1.75 (0.69–4.43)	1.77 (0.70–4.48)
Supplementary education	**2.94** (**1.14–7.56)**	**3.41** (**1.24–9.34)**	**3.36** (**1.12–10.0)**	**3.37** (**1.12–10.1)**
Bachelor	**2.52** (**1.28–4.97)**	**2.81** (**1.35–5.85)**	**3.07** (**1.39–6.75)**	**3.08** (**1.40–6.80)**
Master	**2.67** (**1.18–6.05)**	**2.92** (**1.22–6.97)**	**3.24** (**1.27–8.23)**	**3.25** (**1.27–8.27)**
Occupational status				
Unemployed	1	1	1	1
Employed	1.08 (0.61–1.89)	1.17 (0.65–2.12)	1.35 (0.71–2.55)	1.35 (0.71–2.55)
Benefits during rehab				
Partial sick leave	1	1	1	1
Full sick leave	**0.49** (**0.34–0.70)**	0.85 (0.57–1.27)	0.95 (0.62–1.45)	1.02 (0.65–1.59)
Work assessment allowance	**0.05** (**0.02–0.09)**	**0.07** (**0.04–0.14)**	**0.09** (**0.05–0.18)**	**0.10** (**0.05–0.19)**
Other	**0.23** (**0.13–0.42)**	**0.31** (**0.17–0.58)**	**0.28** (**0.14–0.58)**	**0.29** (**0.14–0.61)**
WAS before		**1.36** (**1.24–1.48)**		**1.57** (**1.41–1.75)**
WAS after			**1.52** (**1.39–1.66)**	
WAS difference				**1.47** (**1.33–1.63)**
Explained variance (*R*^2^)	0.18	0.23	0.29	0.29

Statistically significant values are bold.

All multivariate regression models were adjusted for age, gender, and the other variables listed in the table.

## Discussion

In the present study, we analyzed the WAS both at the start and end of rehabilitation, as well as the change in WAS over the course of rehabilitation. WAS at the start of rehabilitation may be considered a characteristic or state of each individual that affects the chance of RTW even before rehabilitation begins, whereas rehabilitation programs are intended to improve the work ability of the participants and thereby increase their chances of RTW. It was therefore important to examine whether improvements in WAS during rehabilitation would predict RTW, despite the expected collinearity between WAS at the start of rehabilitation and changes in WAS during rehabilitation (e.g., participants with high WAS at the start of rehabilitation have less potential for improving WAS during rehabilitation).

The main finding was a positive association between WAS both at the start and end of rehabilitation and full RTW 12 months later. The best models included either the WAS at the end of rehabilitation (model 3) or both WAS at the start and the change in WAS during rehabilitation (model 4). The chance of full RTW was therefore affected by both the state of the participants before engaging in occupational rehabilitation and the ability of the programs to improve their work ability during rehabilitation. The importance of these factors appeared similar, as the odds ratios were 1.57 and 1.47. This indicated that a one-point increase in WAS (on a 0–10 scale) was associated with a 47%–57% increase in the odds of full RTW.

Furthermore, those who did not achieve full RTW reported, on average, markedly lower improvements in WAS (0.5) compared with those who achieved full RTW (1.4).

The clear association between the self-rated WAS and full RTW is consistent with previous research (13, 18–20). Ståhl et al. (13) reported that the WAS was significantly associated with return to work 6 months after assessment, whereas clinician-administered work ability assessments did not predict return to work. This finding emphasizes the importance of self-assessments and may be explained by the multifactorial causes of sick leave that clinicians cannot identify, even through extended health examinations. These results also highlight the significant potential of WAS as a tool in occupational rehabilitation to assess needs and monitor progress during rehabilitation. Even relatively small improvements in WAS were associated with higher chances of future work participation, and WAS is easy to administer. However, when assessing needs, additional assessments are obviously needed to identify which factors are important to address during rehabilitation. These factors relate to individual resources; however, work ability is a relative concept that also depends on specific factors at the workplace. Most participants probably understand the WAS as an assessment of specific work ability, i.e., they compare their work ability with the demands of their present job or previous employment, which allows rehabilitation professionals to address such factors.

Ahlstrom et al. (11) reported that the WAS performed nearly as well as the full version of the Work Ability Index, which may seem surprising given that the full version contains more detailed information about the sick-listed individual, such as the number of diseases, previous sick leave, and other factors. However, self-assessed work ability may capture all the individual factors that influence the capacity to work (2, 21, 22). Sick-listed patients may be better equipped to weigh the importance of these elements—such as workplace conditions, personal resources, and private and contextual circumstances—than medical doctors or caseworkers.

It may not be trivial for any individual to rate their own work ability. About 20–30% of the participants in our study scored higher at the start compared to the end of rehabilitation. This is often explained by clinicians as a reality check that occurs during rehabilitation: these participants exaggerate their work ability before rehabilitation. During rehabilitation, they test their own physical, mental, emotional, and social resources in social settings with other sick-listed individuals (23, 24), gradually developing a more realistic understanding of their capabilities. Such an interpretation is in line with the findings of the present study, in which the WAS at the end of rehabilitation seemed to be a better predictor of return to work than the WAS at the start of rehabilitation.

WAS at the start of rehabilitation was also associated with full RTW, and it should be noted that participants who received work assessment allowance reported the lowest WAS. This finding is consistent with results reported by Skinnes et al. (2), who also reported substantially lower WAS values 12 months after rehabilitation for this group. Work assessment allowance is granted after 1 year of sick leave benefits. Hence, it is not surprising that recipients reported lower WAS than sick leave beneficiaries, as they had not succeeded in returning to work during the first year of sick-listing. In contrast, sick-listed participants—especially those on partial sick leave—reported higher WAS, consistent with their capacity to work partially. The outcome used in the present study was full RTW, which was achieved by only 5%–6% of those receiving work assessment allowance; however, our data did not permit an analysis of part-time work for this group of beneficiaries. Type of benefit was also a strong predictor of full RTW, which was not surprising, as it reflects both the duration of sick leave and, to some extent, occupational status. Some of those receiving the work assessment allowance have been on sick leave for several years and have also lost their job. However, WAS remained a predictor of full RTW when controlling for these factors.

Gould et al. (9) classified the WAS as poor (scores 0–5), moderate (scores 6–7), good (scores 8–9), and excellent (score 10). In our study, most WAS values ranged between 3 and 6, corresponding to poor to moderate work ability. This level of work ability may be sufficient to initiate a process toward work participation. However, it remains unclear whether work participation 1 year later was associated with further improvements in work ability occurring before or after re-entry into active work.

### Strengths and limitations

A major strength of the present study was the use of register data to measure full RTW. Thus, missing data were avoided when measuring the outcome measure. Missing data could not be entirely avoided in the questionnaires: 24 respondents (2.7%) did not answer all the questions related to sociodemographic factors. Missing data for these variables and for the main “exposure” variable—the WAS—were not imputed, as it was highly suspected that these data were not missing at random.

Another strength was the relatively large number of participants from four different rehabilitation clinics. It should therefore be expected that the findings related to work ability scores are likely applicable and interpretable across similar occupational rehabilitation programs. It remains unclear whether the validity of these results extends beyond rehabilitation participants.

RTW was not measured directly, but it was assumed that not receiving health-related benefits implied RTW. In Norway, it is very uncommon for individuals with significant health problems and reduced ability to work to remain without such benefits. The term “full RTW” was used because it was not possible to determine whether graded or partial work participation was achieved from data on benefits. While graded sick leave benefits suggest partial work participation, work assessment allowance can also be received during gradual return to work. Therefore, it was decided to use only full return to work as an outcome measure, even though this underestimated total work participation. “Full RTW” was measured at 12-month follow-up for an entire month, which reflected sustained RTW, but it was not known whether RTW would be sustained for several months.

A limitation was that data on other factors that could be potential predictors of RTW were not available. RTW may be a complex process influenced by several other factors, such as specific health problems (25, 26), working environment (27–29), and additional personal issues (25–28). The attending clinics did not provide data on diagnosis for the present study, which was another limitation. Therefore, it remains unclear whether the associations between work ability and RTW were similar for participants with musculoskeletal and mental diagnoses. However, in many cases, participants are referred to occupational rehabilitation programs with more than one health condition (25, 30), and it is not always clear which condition contributes to work-related disability. In particular, the combination of musculoskeletal and mental health problems is common among long-term sickness absentees receiving occupational rehabilitation (30).

Physical and psychosocial working environment issues were not assessed before rehabilitation, but they were addressed during the rehabilitation program. For instance, employers were routinely contacted by rehabilitation professionals and often also by the participants to discuss challenges and possibilities for RTW. For some participants, it was decided during the rehabilitation program not to plan a return to the same workplace because of working environment barriers. However, information on these decisions was not available in the occupational rehabilitation registry.

### Practical implications

The single-item WAS may be used in clinical practice to assess rehabilitation needs at the start of rehabilitation and to evaluate and document improvements during the program. Poor work ability, low education, and longer duration of sickness absence before rehabilitation may indicate a need for stronger and more intensive rehabilitation efforts compared with individuals with moderate work ability. Furthermore, it is difficult to assess the performance of occupational rehabilitation programs that aim for work participation, which may occur after the program ends. Both clinicians and policymakers should also focus on goals that are achieved during rehabilitation and that strongly predict future work participation, such as WAS.

## Conclusions

The strong association between improvements in the WAS during rehabilitation and full RTW 1 year later demonstrates the importance of strengthening the physical, mental, emotional, and social resources of the participant that may improve their work ability during rehabilitation. Even seemingly small improvements in work ability may initiate a process that ultimately leads to work participation. Future studies should examine whether work participation 1 year later is associated with changes in work ability that occur before or after re-entry into active work.

## Data Availability

The raw data supporting the conclusions of this article will be made available by the authors, without undue reservation.
